# Mitochondrial transcription and translation: overview

**DOI:** 10.1042/EBC20170102

**Published:** 2018-07-20

**Authors:** Aaron R. D’Souza, Michal Minczuk

**Affiliations:** MRC Mitochondrial Biology Unit, University of Cambridge, Cambridge, U.K.

**Keywords:** mitochondria, trascription, translation

## Abstract

Mitochondria are the major source of ATP in the cell. Five multi-subunit complexes in the inner membrane of the organelle are involved in the oxidative phosphorylation required for ATP production. Thirteen subunits of these complexes are encoded by the mitochondrial genome often referred to as mtDNA. For this reason, the expression of mtDNA is vital for the assembly and functioning of the oxidative phosphorylation complexes. Defects of the mechanisms regulating mtDNA gene expression have been associated with deficiencies in assembly of these complexes, resulting in mitochondrial diseases. Recently, numerous factors involved in these processes have been identified and characterized leading to a deeper understanding of the mechanisms that underlie mitochondrial diseases.

## Introduction

Mitochondrial gene expression is central to maintaining cellular homoeostasis. The control of mitochondrial gene expression is unique in that its components have dual origins in the mitochondria (all RNAs) and nucleus (all protein factors). The regulation of the synthesis and degradation of mitochondrial (mt-) RNAs determines steady-state levels of mitochondrially encoded proteins allowing fine control of the mitochondrial energy metabolism. Therefore, the cell can adapt to changing environmental stresses and satisfy changing cellular energy demands. Defects in the mitochondrial gene expression can lead to respiratory chain dysfunction resulting in a multi-system disease phenotype, predominantly affecting muscular and neuronal tissues.

**Figure 1:**
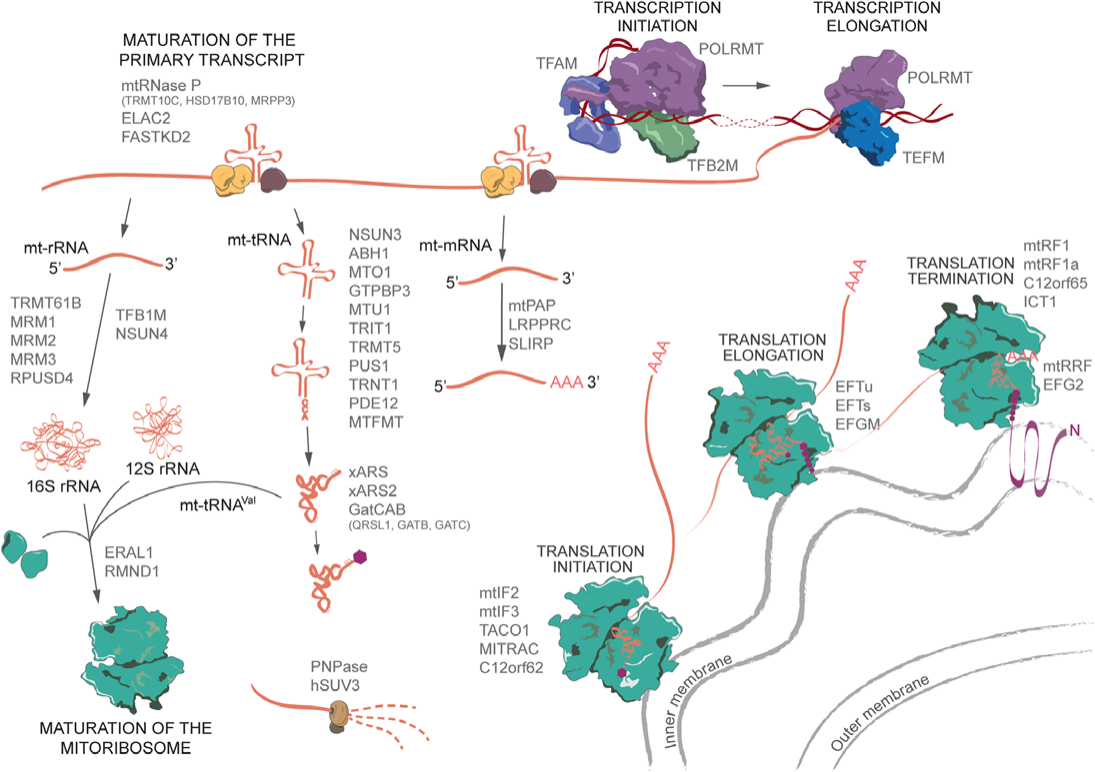
Overview of human mitochondrial transcription, RNA processing and translation The list of proteins mentioned in the figure are biased towards those that are associated with mitochondrial diseases, as explained in the article by Boczonadi et al. [1]. Aminoacyl-tRNA synthetases are abbreviated as xARS and xARS2.

The mammalian mitochondrial genome is highly condensed as far as genetic information is concerned. The mitochondrial genome encodes 2 rRNAs, 22 tRNAs and mRNAs for 13 polypeptides of the oxidative phosphorylation (OxPhos) system. In some cases, the reduction of the mitochondrial genome has led to overlapping genes (*MT-ATP8/6, MT-ND4/4L* and mt-tRNA^Tyr^/mt-tRNA^Cys^). The entire mitochondrial genome is transcribed from both the strands as long polycistronic transcripts. These strands are named heavy (H) or light (L) based on their buoyancy in caesium chloride density gradients. The long polycistronic transcripts require multiple processing steps before individual RNA species become functional. After endonucleolytic cleavage of the primary transcript, the ribosomal RNAs undergo chemical modifications before it can function correctly within the mitoribosome, the tRNAs also undergo a large number of chemical modifications, in addition to further polymerization and aminoacylation, and the mRNAs are differentially polyadenylated. Finally, the mRNAs, tRNAs and the assembled mitoribosome come together in the translation apparatus where translational factors direct the progression of translation (Figure 1).

In this article, we briefly overview the key stages of mitochondrial gene expression in humans, providing a useful basis for the article by Boczonadi et al. that deals with human diseases resulting from the defects in this pathway [1]. The main goal of our article is to present the basic mitochondrial function of protein factors that have been associated with mitochondrial disease, therefore, some proteins known to be involved in mitochondrial transcription and translation might have not been described in this brief overview.

## Mitochondrial transcription

Transcription of the mitochondrial genome originates in the major non-coding region containing the L-strand (LSP) and H-strand (HSP) promoters. The light strand promoter controls the transcription of eight of the tRNAs and the *MT-ND6* gene. On the heavy strand, two H-strand two-promoter systems have historically been proposed, where HSP1 transcription produces a transcript containing tRNA^Phe^, tRNA^Val^ and the two rRNAs (12S and 16S), while transcription from HSP2 generates a transcript that spans almost the entire genome [2–4]. This two-promoter model of H-strand transcription was proposed to explain the high abundance of the two rRNAs. However, more recent animal models [5] and *in vitro* [6] experiments suggest that heavy strand transcription is under the control of a single promoter and that the difference in rRNA abundance may be a consequence of differential turnover.

### Transcription initiation

Transcription in human mitochondria is driven by a DNA-dependant RNA polymerase called POLRMT, which is structurally similar to RNA polymerases in T3 and T7 bacteriophages [7,8]. This includes high sequence homology to the C-terminal catalytic core of the enzyme [9]. At the N-terminal domain, POLRMT also contains two pentatricopeptide repeat (PPR) domains, commonly found in RNA-associated proteins, where they are required for site-specific interactions [7,10]. In contrast with bacteriophage polymerases, which can recognize promoter regions without auxiliary proteins, additional factors are required to perform this function by POLRMT. The initiation of transcription requires the association of POLRMT with mitochondrial transcription factor A (TFAM) and mitochondrial transcription factor B2 (TFB2M). TFAM is a DNA-binding protein, which, in addition to transcription activation, also packages DNA in the nucleoid [11]. TFB2M was produced as a result of a gene duplication event. TFB1M, the other product of this duplication event, is a ribosomal RNA methyltransferase (see below). Although TFB2M also contains a rRNA methyltransferase domain, the key function of this protein is DNA melting during the initiation of transcription [12–16]. Recent evidence shows that in the transcription initiation complex, at both the HSP and LSP, TFAM, bound to DNA, recruits POLRMT to the promoter via its N-terminal extension. TFB2M modifies the structure of POLRMT to induce opening of the promoter [14,17,18].

### Transcription elongation

POLRMT requires an additional transcription elongation factor (TEFM) for the elongation stage [19]. Recombinant TEFM strongly promotes POLRMT processivity as it stimulates the formation of longer transcripts *in vitro* [20]. Also, depletion of TEFM in living cells leads to a reduction in promoter–distal transcription elongation products [19]. Transcription from LSP is often prematurely terminated around the conserved sequence block 2 (CSB2) of the major non-coding region of the mitochondrial genome. The short RNA molecule produced has been suggested to play a key role in priming DNA replication as multiple RNA to DNA transition sites clustering around CSB2 [21,22] (see also the article by Maria Falkenberg [23]). Stimulation of POLRMT processivity by TEFM prevents the formation of G-quadraplexes that inhibit the progression of the elongation complex at CSB2 [20,24]. The capability of TEFM to abolish premature transcription termination has been proposed to function as the switch from replication to the transcription of the LSP-derived primary transcript [24]. Recent structural work showed that TEFM contains a pseudonuclease core that forms a ‘sliding clamp’ around the mtDNA downstream of the transcribing POLRMT, interacting with POLRMT via its C-terminal domain [25].

### Transcription termination

The mechanism of termination of HSP transcription is still unclear. It was previously suggested that mitochondrial termination factor 1 (MTERF1) bends the mtDNA connecting the HSP1 promoter site and its apparent tRNA^Leu(UUR)^ termination site. MTERF1 would then induce transcription termination through base flipping and DNA unwinding [26–28]. This model was originally proposed to explain the 50-fold higher abundance of mitochondrial rRNAs [27]. However, more recent evidence contradicts this hypothesis. Studies in MTERF1 knockout mice do not show an effect on rRNA steady-state levels [5]. Their increase in abundance is probably a product of increased stability rather than due to the presence of a different promoter. Moreover, it was also recently shown that transcription from the LSP is prematurely terminated by MTERF1 at the 3′-end of the mt-rRNA coding sequence. Binding of MTERF1 to this site prevents the replication fork from progressing into the mt-rRNA genes while they are being transcribed, whilst also preventing transcription of the antisense sequence of the rRNA [5,29,30].

## Maturation of the primary transcript

Transcription from the heavy and light strand promoters produces long polycistronic transcripts. The mt-rRNA coding sequences and most of the protein coding sequences are separated by mt-tRNAs. Endonucleolytic excision of these mt-tRNAs releases the mRNAs and rRNAs – a concept known as the ‘tRNA Punctuation Model’ [31,32]. The processing of mt-tRNAs from the primary transcript is performed by RNase P and RNase Z at the 5′- and 3′-end respectively. Unlike previously characterized cytoplasmic and bacterial RNase P enzymes, which contain a catalytic RNA subunit, mammalian mitochondrial RNase P is an entirely proteinaceous heterotrimeric endonuclease. This enzyme is composed of a tRNA m^1^R9 methyltransferase, TRMT10C (MRPP1), a member of the short-chain dehydrogenase/reductase (SDR) family, SDR5C1 (HSD17B10, MRPP2), and a protein with homology to PiIT N-terminus (PIN) domain-like metallonucleases, PRORP (MRPP3) and cleaves the primary transcript at the 5′-end of tRNAs [33]. ELAC2 is an endonuclease that executes 3′-end maturation of both mitochondrial and nuclear pre-tRNAs [34–36].

The ‘tRNA punctuation model’ does not explain all the primary transcript cleavage events, as not all mRNAs are immediately flanked by tRNAs. Various Fas-activated serine/threonine kinase (FASTK) proteins have been shown to be required for mtRNA stability and the processing of precursors, especially the non-canonical cleavage sites. They all contain a conserved nuclease fold (RAP domain); however, endonucleolytic activity has not been shown for any of the FASTK proteins [37]. For example, depletion or knockout of FASTKD2 leads to the accumulation of various cleavage precursors, especially 16 rRNA and ND6 mRNA [38,39]. Additionally, FASTK has been implicated in MT-ND6 maturation and stability, and FASTKD5, similar to FASTKD4, regulates the maturation of those precursor RNAs that cannot be processed by RNase P and ELAC2 [39,40]. Furthermore, cross-linking immunoprecipitation (CLIP)-based analysis of FADTKD2 binding sites identified 16S rRNA and ND6 as its targets [38]. Recently, FASTKD4 was shown to be required for the stable expression of several mt-mRNAs, whereas FASTKD1 had the opposite effect on the stability of the MT-ND3 mt-mRNA. Interestingly, depletion of both FASTKD1 and FASTKD4 also caused a loss of MT-ND3, suggesting that the loss of FASTKD4 is epistatic [37]. Moreover, FASTKD4 has been suggested to be responsible for the cleavage of the MT-ND5-CYB precursor. A detailed characterization of how the FASTK proteins regulate the mitochondrial transcriptome is likely to be a subject of intense study in the near future.

### mRNA maturation and stability

After excision from the primary transcript, all mRNAs, except MT-ND6, undergo 3′ polyadenylation. Polyadenylation in mitochondria is performed by a homodimeric polyadenylic acid RNA polymerase (mtPAP) [41–43]. Seven of thirteen mt-mRNAs do not contain a 3′ stop codon. In these cases, 3′ adenylation completes these stop codons and thus, the open reading frame. Polyadenylation of bacterial transcripts generally mark them for degradation, whereas addition of poly(A) tails to eukaryotic, nuclear-encoded mRNA is necessary for their stability. However, in mammalian mitochondria the effect of polyadenylation on steady-state levels is mRNA-specific. For example, deadenylation decreases complex IV mt-mRNA and increases complex I mt-mRNA levels [41,44–46].

The stability of HSP-derived mitochondrial transcripts is regulated by leucine-rich penticopeptide rich domain containing protein (LRPPRC) [47]. Loss of LRPPRC reduces the steady-state levels of mRNAs whilst not affecting rRNAs and tRNAs, consequently leading to a translation defect and loss of respiratory complexes [48–51]. The presence or absence of LRPPRC in the mitochondria correlates with the level of mt-mRNA polyadenylation [52,53]. As such, LRPPRC mouse knockout models display a loss in HSP-derived transcripts, loss of poly(A) tails and a severe translational defect [50]. More recent data show that LRPPRC is a mt-mRNA chaperone that relaxes secondary structures, therefore, facilitating RNA polyadenylation and coordinated mitochondrial translation [54]. Following translocation into mitochondria, LRPPRC forms a complex with a stem–loop interacting RNA-binding protein (SLIRP) [55]. Within this complex, SLIRP stabilizes LRPPRC by protecting it from degradation [56], whilst being dispensable for polyadenylation of mtDNA-encoded mRNAs [56]. The LRPPRC–SLIRP complex has also been shown to suppress their degradation of mt-mRNAs [57].

Human mitochondrial RNA decay is mediated by a complex of polynucleotide phosphorylase (PNPase) and human Suv3 protein (hSuv3) [58]. PNPase is a 3′–5′ exoribonuclease which has been shown to localize to the intermembrane space [59], and also in distinct foci with hSuv3p and mitochondrial RNA [58]. Knockdown of PNPase leads to the increase in the half-life of mitochondrial transcripts and the accumulation of RNA decay intermediates [58]. hSuv3p is an NTP-dependent helicase. The lack of a functioning hSuv3 helicase leads to the accumulation of aberrant RNA species, polyadenylated molecules and degradation intermediates [60]. Recent evidence shows that exposure to the intercalating agent ethidium bromide (EtBr), which disrupts tRNA secondary structure, causes them to be polyadenylated. Subsequent withdrawal of EtBr causes the polyadenylated tRNAs to be rapidly degraded by the PNPase–hSuv3 degradosome [61]. Knockdown of PNPase leads to lengthening of the poly(A) tails due to inhibited tRNA turnover [61]. Controversially, the localization of PNPase in the intermembrane space has led to implications that it plays a role in the import of endogenous RNA into mitochondria. However, various pieces of evidence suggest that this is not the case and that it primarily functions in the RNA degradosome [62].

### tRNA maturation

The mt-tRNAs undergo extensive post-transcriptional maturation including chemical nucleotide modifications and CCA addition at the 3′-end deadenylation. One of the key tRNA positions of chemical modification is the ‘wobble’ base (position 34) at the anticodon of the tRNAs. During translation, the appropriate amino acyl-tRNA is positioned in the mitoribosome through the accurate recognition of a cognate mRNA codon. However, since, many codons code for the same amino acid, the first position of the tRNA anticodon is chemically modified to facilitate non-Watson–Crick base pairing, therefore expanding codon recognition during mitochondrial translation. Some of the enzymes involved in modifying this position include: NSUN3 and ABH1 which are required for the introduction of 5-formylcytosine at the wobble position (f^5^C34) of mt-tRNA^Met^ [63–66], MTO1 and GTPBP3 are required for the biogenesis of 5-taurinomethyluridine (τm^5^U) [67,68], and MTU1 (TRMU) which catalyses the 2-thiolation of 5-taurinomethylridine to form τm^5^s^2^U of a subset of mt-tRNAs [69,70].

In addition to the modification of the wobble position, position 37 downstream of the anticodon is also frequently chemically modified in order to facilitate stable codon–anticodon interactions and, therefore, increasing accuracy and fidelity of mitochondrial translation. Examples of enzymes that are responsible for modifying mt-tRNA position 37 include TRIT1 responsible for the introduction of an isopentenyl group onto *N*^6^ of 37 adenine (i^6^A37) in a small subset of mt-tRNAs [71] or TRMT5 which introduces *N*^1^-methylation of the 37 guanosine (m^1^G37) [72].

Pseudouridine (Psi), the most common RNA modification, is often referred to as the fifth nucleotide. It is a structural isomer of uridine produced by a rotation around the N3–C6 axis. Psi is generally associated with a stabilizing role, by providing structural rigidity to RNA molecules regardless of sequence or structure, and has been detected in several mt-tRNAs. PUS1 is a pseudouridine synthetase which modifies U27 and U28 on mt-tRNAs [73,74]. Recently, a pseudouridine synthetase, RPUSD4, was characterized as introducing pseudouridine at position 39 of tRNA^Phe^ [75]. Other putative PUSs have been identified as necessary for mitochondrial translation, including RPUSD3 and TRUB2; however, their exact mtRNA targets remain to be further characterized [76,77].

The CCA found at the 3′-end of all mature mt-tRNAs is not encoded by mtDNA and is instead post-transcriptionally synthesized by tRNA-nucleotidyltransferase 1 (TRNT1): TRNT1 does not require a template sequence, instead preferentially selecting CTP and ATP for polymerization [78]. Similarly, a non-encoded 5′ guanine on mt-tRNA^His^ is post-transcriptionally added by 3′–5′ polymerase activity probably provided by THG1L [79]. The 3′ ends of several mt-tRNAs undergo spurious, mistargeted adenylation precluding correct aminoacylation at the 3′-end (see below). A 3′–5′ exonuclease, PDE12, is required for the removal of these spurious poly(A) tails [45,80].

### tRNA aminoacylation

Mitochondrial translation requires that each tRNA is charged with the cognate amino acid. This process is mediated by the mitochondrial aminoacyl-tRNA synthetases (ARS2s), which are encoded by nuclear genes. Of these, 17 ARS2s are unique to the mitochondria, while GARS (Glycyl-tRNA synthetase) and KARS (Lysyl-tRNA synthetase) are encoded by the same loci as the cytoplasmic enzymes, with the mitochondrial isoforms being generated by alternative translation initiation (GARS) [81] or alternative splicing (KARS) [82]. Interestingly, glutaminyl mt-tRNA (mt-tRNA^Gln^) is aminoacylated by an indirect pathway, in which it is first charged with glutamic acid (Glu) by mitochondrial glutamyl-tRNA synthetase (EARS2), after which the Glu-mt-tRNA^Gln^ is transamidated into Gln-mt-tRNA^Gln^, using free glutamine as an amide donor [83]. This latter conversion is performed by GatCAB, the glutamyl-tRNA^Gln^ amidotransferase protein complex, which consists of three subunits: GatA (QRSL1), GatB (GATB) and GatC (GATC) [84].

## Mitochondrial ribosome: structure and assembly

The mitoribosome consists of a large 39S subunit (mtLSU) and a small 28S (mtSSU) subunit. Compared with the bacterial ribosome, the mammalian mitoribosome has reduced rRNA components. To compensate for this, 36 mitochondria-specific proteins have been recruited to the ribosome, primarily found at the periphery of the complex surrounding a highly conserved catalytic core [85–89]. In the bacterial ribosome, a 5S rRNA acts as a scaffold interconnecting the LSU, SSU and the tRNAs in the intersubunit space. However, recent structures of the mitoribosome instead identified the recruitment of a mitochondrially encoded tRNA to this site (tRNA^Val^ in humans and rat, tRNA^Phe^ in porcine and bovine mitochondria) [88,90,91].

The maturation of the mitoribosome requires the post-transcriptional processing of the catalytic rRNA in addition to the import and assembly of about 80 nuclear-encoded proteins (MRPL and MRPS proteins). As for other ribosomes, both the small subunit and the large subunit rRNA undergo chemical nucleotide modifications. These modifications include base methylations, 2′-*O*-ribose methylations and pseudouridylation, with several enzymes responsible for these modifications having been identified (TRMT61B [92]; TFB1M [93]; NSUN4 [94]; MRMs [95]; RPUSD4 [76]; reviewed in [96]). For example, the A-loop region of the 16S rRNA is methylated at position U1369 and G1370 (human mtDNA numbering). This site directly interacts with the aminoacyl-tRNA [88,97]. U1369 is methylated by MRM2, which has been shown to interact with the mtLSU. Depletion of the protein leads to defective biogenesis of the mtLSU and consequently, a deficiency in translation [97]. Also, several protein factors not directly involved in rRNA modification have been identified to coordinate the assembly of the mitoribosome reviewed in [96]. For example, ERAL1, a homolog of the bacterial Era protein that belongs to the conserved family of GTP-binding proteins, has been proposed to act as an RNA chaperone that stabilizes 12S mt-rRNA during mtSSU assembly [98].

Many of the proteins involved in mitoribosome assembly and the post-transcriptional processing of the nascent transcript, including FASTK proteins, ELAC2 or RNase P (see above) are found in distinct foci called mitochondrial RNA granules (MRG). This compartmentalization has been proposed to facilitate more efficient and accurate gene expression [40,99,100]. It has been suggested that an integral inner membrane protein, RMND1, stabilizes and anchors the mitochondrial ribosome at the inner membrane, adjacent to MRGs where the mRNAs are produced and processed [101]. However, the exact function and mechanism of this protein is still unclear.

## Translation

Mitochondrial translation is fully dependent on various nuclear-encoded regulatory proteins. In the mammalian mitochondria, the mitochondrial initiation factors, mtIF2 and mtIF3, control the initiation of translation [102]. During initiation, mtIF3 positions the AUG or AUA initiation codons of the mRNA at the peptidyl (P) site in the mtSSU and prevents the premature association of the mtLSU and mtSSU [103–105]. As in all protein synthesis systems, translation in mitochondria is initiated with a methionine residue. However, mitochondria differ in that only a single tRNA^Met^ is used for both initiation and elongation. Discrimination, instead, is achieved through a post-transcriptional modification, with the aminoacylated initiator mt-tRNA^Met^ being subjected to formylation of methionine (fMet), thereby increasing its affinity for mtIF2 [106]. mtIF2 directs the association of the fMet-tRNA^Met^ with the mRNA, and guides the assembly of the mitochondrial monosome and the initiation of translation [107,108].

Translation in mammalian mitochondria differs from that of the cytoplasm or that of the yeast mitochondria in part due to the general absence of 5′-untranslated regions (UTRs) on mRNAs, gene-specific RNA *cis*-acting regulatory elements and introns. In yeast, a 5′-UTRs allow mRNA-specific, translational activators to bind and direct the mRNA into the mitoribosome for translation. However, in mammalian mitochondria, such mRNA regulatory elements have not been identified. Hence, alternative mechanisms are in place for the regulation of translation. Unlike UTR-based regulation, these protein factors have to bind directly to the mitochondrial transcript and affect gene expression. For example, various protein factors such as TACO1, MITRAC or C12orf62 have been recruited to modulate the translation of complex IV subunit CO1. Absence of any of these protein leads to a complex IV deficiency [109–111].

Elongation of translation is mediated by mitochondrial elongation factors, EFTu (TUFM), EFTs (TSFM) and EFGM (GFM1) [112,113]. In elongation, EFTu forms a complex with GTP and aminoacyl tRNA. It directs the tRNA to the acceptor (A) site where the tRNA base pairs with the mRNA at the codon–anticodon site. The hydrolysis of GTP catalyses peptide bond formation. EFTu is released and the GTP:EFTu complex is re-established by EFTs [114]. EFG1-mt causes the release of the deacetylated tRNA from the P-site, translocates the peptidyl-tRNAs from the A and P site to the P and exit (E) site, also causing the mRNA to move along by one codon.

Termination of mitochondrial translation is finally triggered by the presence of a stop codon at the A-site. Four mitochondrial proteins with homology to ribosome release factors have been identified in humans, including mtRF1, mtRF1a, C12orf65 and ICT1. These factors are characterized by the presence of a tripeptide GGQ motif that confers peptidyl-tRNA hydrolase activity [115,116]. Structural analysis of mtRF1 suggested that it is capable of recognizing the UAA and UAG stop codons, targeting ribosomes with a vacant A-site [117,118]. However, it does not exhibit release activity *in vitro* [119]. mtRF1a catalyses the hydrolysis of peptidyl tRNA at the UAA and UAG stop codons [120]. mtRF1a has been proposed to be sufficient for the termination of translation of all 13 mtDNA-encoded polypeptides, despite the mRNAs for MT-CO1 and MT-ND6 lacking the UAA and UAG stop codons at the end of the open reading frame (ORF). Instead, these ORFs contain in-frame AGA and AGG as the last codons respectively. AGA and AGG are used to encode Arg according to the universal genetic code; however, they are not used for this purpose in any of the mitochondria ORFs. Fine mapping of the termination codons of the mRNAs showed that these two mRNAs terminate at the UAG stop codon possibly created as a result of a −1 frameshift of the mitoribosome [121,122]. Initially, ICT1 was suggested as the protein that performed the termination function at the AGA and AGG codons. However, neither ICT1 nor C12orf65 release factor homologues containing the specific domains required for UAA and UAG stop codon recognition [116]. Recent evidence also suggests that ICT1 is capable of inducing hydrolysis of the peptidyl tRNAs in stalled mitoribosomes [119,123]. Since ICT1 is incapable of performing the peptidyl hydrolase activity, where the RNA template extends 14 nucleotides beyond the A-site, as is the case in MT-CO1 and MT-ND6, ICT1 may not be directly involved in the termination of translation of these two mRNAs [124,125].

Finally, after the release of the polypeptide, mitochondrial ribosomal recycling factors, mtRRF and EFG2 (also known as RRF2M, a homologue of EFGM) catalyse the release of the mRNAs, deacetylated tRNAs and the ribosomal subunits [126,127].

## Concluding remarks

Diseases affecting mitochondrial transcription and translation, as described in the article by Boczonadi et al. [1], can have multi-systemic and severe manifestation. The development of novel, treatments of these mitochondrial diseases can be made more effective through a deeper understanding of the underlying mechanisms that cause them.

## Summary

- Progression of mitochondrial transcription and translation requires the sequential recruitment of different, nuclear-encoded initiation, elongation and termination factors.
- Almost the entire mitochondrial genome is transcribed as long polycistronic transcripts.
- Maturation of the transcripts requires endonucleolytic cleavage, but not all mRNAs are produced through RNase P and RNase Z function.
- Mitochondrial mRNA steady-state levels are mainly controlled post-transcriptionally.
- The role mitochondrial mRNA polyadenylation is not fully understood.
- Mitochondrial tRNAs undergo extensive chemical modifications, including the addition and removal of nucleotides during their maturation.
- Aminoacyl tRNA-synthetases charge tRNAs with their cognate amino acid, many of which are unique to the mitochondria.
- Mammalian mitoribosomes differ considerably from other ribosomes as far as architecture and composition are concerned, with the key differences being the reversed protein:RNA mass ratio, incorporation of mtDNA-encoded structural tRNA and many novel, mitochondria-specific protein components.
- The assembly of the mitoribosome assembly pathway is likely to be considerably different from its bacterial counterpart, implying the presence of mitochondria-specific regulatory factors.

## Abbreviations

ARS2: aminoacyl-tRNA synthetase
CSB2: conserved sequence block 2
FASTK: Fas-activated serine/threonine kinase
HSP: H-strand promoter
hSuv3: human Suv3 protein
LRPPRC: leucine-rich penticopeptide rich domain containing protein
LSP: L-strand promoter
MTERF1: mitochondrial termination factor 1
PNPase: polynucleotide phosphorylase
SLIRP: stem–loop interacting RNA-binding protein
TRNT1: tRNA-nucleotidyltransferase 1
